# Spontaneous Centralization of Control in a Network of Company Ownerships

**DOI:** 10.1371/journal.pone.0080303

**Published:** 2013-12-06

**Authors:** Sebastian M. Krause, Tiago P. Peixoto, Stefan Bornholdt

**Affiliations:** Institut für Theoretische Physik, Universität Bremen, Bremen, Germany; Max Planck Institute for the Physics of Complex Systems, Germany

## Abstract

We introduce a model for the adaptive evolution of a network of company ownerships. In a recent work it has been shown that the empirical global network of corporate control is marked by a central, tightly connected “core” made of a small number of large companies which control a significant part of the global economy. Here we show how a simple, adaptive “rich get richer” dynamics can account for this characteristic, which incorporates the increased buying power of more influential companies, and in turn results in even higher control. We conclude that this kind of centralized structure can emerge without it being an explicit goal of these companies, or as a result of a well-organized strategy.

## Introduction

The worldwide network of company ownership provides crucial information for the systemic analysis of the world economy [1], [2]. A complete understanding of its properties and how they are formed has a wide range of potential applications, including assessment and evasion of systemic risk [3], collusion and antitrust regulation [4], [5], market monitoring [6], [7], and strategic investment [8]. Recently, Vitali et al [9] inferred the network structure of global corporate control, using the Orbis 2007 marketing database [10]. Analyzing its structure, they found a tightly connected “core” consisting of a small number of large companies (mostly financial institutions) which control a significant part of the global economy. A central question which arises is what is the dominant mechanism behind this centralization of control. The answer is not obvious, since the decision of firms to buy other firms can be driven by diverse goals: Banks act as financial intermediaries doing monitoring for uninformed investors [6], [7], managers can improve their power by buying other firms instead of paying dividends [11], speculation on stock prices, as well as dividend earnings can be a significant source of revenue [11]–[13], and companies can have strategic advantages, e.g. due to knowledge sharing [8], [14], [15]. Another possible hypothesis for control centralization is that managers collude to form influential alliances: Indeed, agents (e.g. board members) often work for different firms in central positions [16]. Although all these factors are likely to play a role, we here investigate a different hypothesis, namely that a centralized structure may arise spontaneously, as a result of a simple “rich-get-richer” dynamics [17], without any explicit underlying strategy from the part of the companies. We consider a simple adaptive feedback mechanism [18] which incorporates the indirect control that companies have on other companies they own, which in turn increases their buying power. The higher buying power can then be used to buy portions of more important companies, or a larger number of less important ones, which further increases their relative control, and progressively marginalizes smaller companies. We show that this simple dynamical ingredient suffices to reproduce many of the qualitative features observed in the real data [9], including the emergence of a core-periphery structure and the relative portion of control exerted by the dominating core. Although this does not preclude the possibility that companies may take advantage and further consolidate their privileged positions in the network, it does suggest that deliberate strategizing may not be the dominating factor which leads to global centralization.

## Model Description

We consider a network of 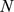 companies, where a directed edge between two nodes 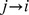 means company 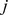 owns a portion of company 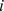. The relative amount of 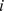 which 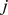 owns is given by the matrix 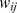 (i.e. the ownership shares), such that 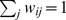. We note that it is possible for self-loops to exist, i.e. a company can in principle buy its own shares. In the following, we describe a model with two main mechanisms: 1. The evolution of the relative control of companies, given a static network; 2. The evolution of the network topology via adaptive rewiring of the edges.

### 1.1 Evolution of control

**Figure 1:**
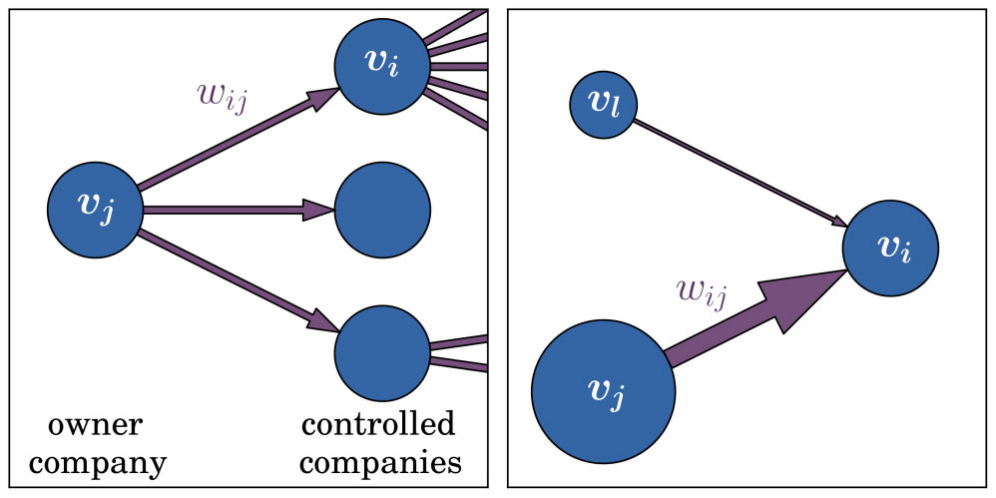
Illustration of control and ownership. Left: The company 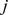 owns portions of three other companies. The relative control over a company 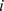 is proportional to the ownership share represented by the weight 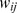. The relative control value 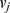 of 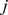 partially inherits the value 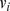 of the owned company 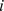. In this way, indirect control is included. Right: The control weights 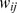 are themselves distributed in a manner which is proportional to the overall relative control of the corresponding controlling company, such that more important companies tend to have bigger shares.

Here we assume that if 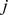 owns 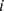, it exerts some influence on 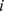 in a manner which is proportional to 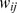. If we let 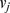 describe the relative amount of control a company 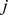 has on other companies, we can write
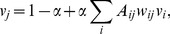
(1)where 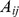 is the adjacency matrix, the parameter 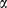 determines the propagation of control and 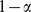 is an intrinsic amount of independence between companies. Eq. 1 can be seen as a weighted version of the Katz centrality index [19], which is one of many ways of measuring the relative centrality of nodes in a directed network, such as PageRank [20] and HITS [21]. It converges for 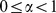 and we enforce normalization with 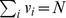. We further assume that the control value 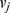 directly affects other features such as profit margins, and overall market influence, such that the buying power of companies with large 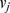 is also increased. This means that the ownership of a company 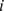 is distributed among the owners 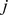, proportionally to their control 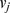, i.e.
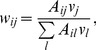
(2)(see Fig. 1). These equations are assumed to evolve on a faster time scale, such that equilibrium is reached before the topology changes, as described in the next section.

### 1.2 Evolution of the network topology

**Figure 2:**
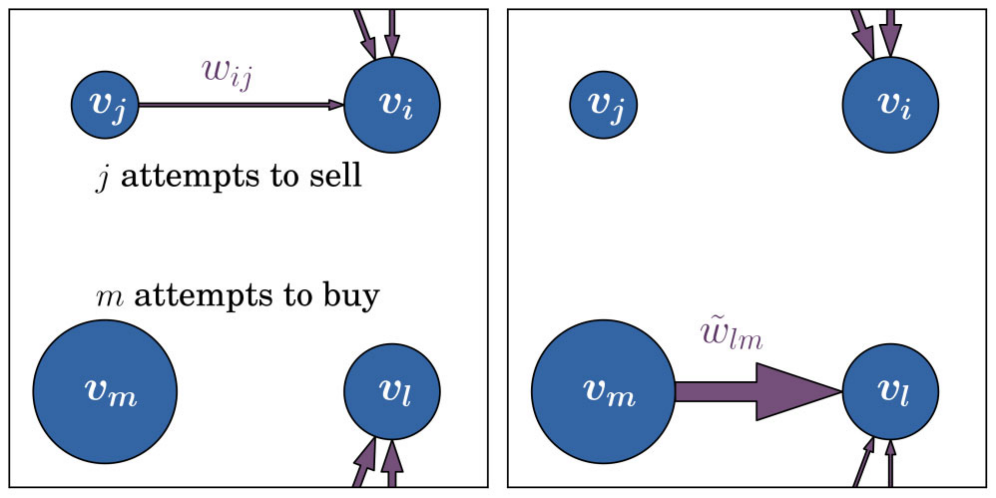
Illustration of the adaptive process. Snapshots before the rewiring (left) and afterwards (right), where the edge 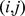 was deleted from the graph (company 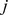 gave up its shares of 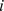), and new edge 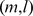 was added (company 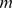 bought shares of company 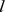). Important links with high values 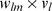 are favored according to the replacement probability of Eq. 3.

Companies may decide to buy or sell shares of a given company at a given time. The actual mechanisms regulating these decisions are in general complicated and largely unknown, since they may involve speculation, actual market value, and other factors, which we do not attempt to model in detail here. Instead, we describe these changes probabilistically, where an edge may be deleted or inserted randomly in the network, and such moves may be accepted or rejected depending on how much it changes the control of the nodes involved. For simplicity, we force the total amount of edges in the network to be kept constant, such that a random edge deletion is always accompanied by a random edge insertion. Such “moves” may be rejected or accepted, based on the change they bring to the 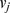 values of the companies involved. If we let 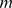 be the company which buys new shares of company 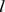, and 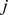 which sells shares of company 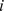, the probability that the move is accepted is
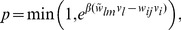
(3)where 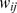 is computed before the move and 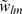 afterwards, and the parameter 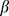 determines the capacity companies have to foresee the advantage of the move, such that for 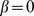 all random moves are accepted, and for 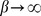 they are only accepted if the net gain is positive (see Fig. 2). Note that in Eq. 3 it is implied that companies with larger control will tend to buy more than companies with smaller control, which is well justified by our assumption that control is correlated with profit and wealth.

The overall dynamics is composed by performing many rewiring steps as described above, until an equilibrium is reached, i.e. the observed network properties do not change any longer. In order to preserve a separation of time scales between the control and rewiring dynamics, we performed a sufficiently large number of iterations of Eqs. 1 and 2 before each attempted edge move. For this we introduced an additional parameter 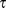 which incorporates separation of time scales in the limit 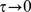, and the exact iterative rules were performed as follows. In each time step we choose one of the two options:

1. With probability 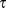, a rewiring move is considered as follows. An edge is chosen at random, where the owner (source of the edge) 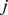 attempts to give up the shares of the owned company (target of the edge) 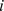, i.e. the edge 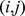 is deleted from the network. Additionally, two non-adjacent companies 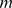 and 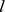 are chosen at random, and 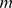 attempts to buy new shares of company 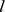, i.e. the edge 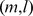 is inserted in the network. If the move is accepted due to Eq. 3 (with the additional requirement that 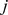 is not the last remaining owner of 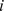), the number of owners of 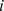 and 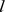 both change. Therefore, all weights 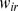 and 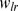 with 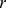 denoting the owners are updated via Eq. 2.
2. With probability 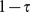, the control values 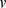 and weights 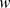 are updated as follows. A company 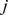 is randomly chosen, its control value 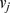 is updated via Eq. 1, and the weights of the owners 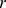 of 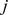, 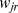, are updated via Eq. 2.

We performed simulations with 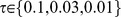 and found, that the results do not differ significantly in this range. Thereafter we used 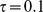 for all simulations presented in this paper, as it is sufficient to separate the time scales.

## Centralization of Control

**Figure 3:**
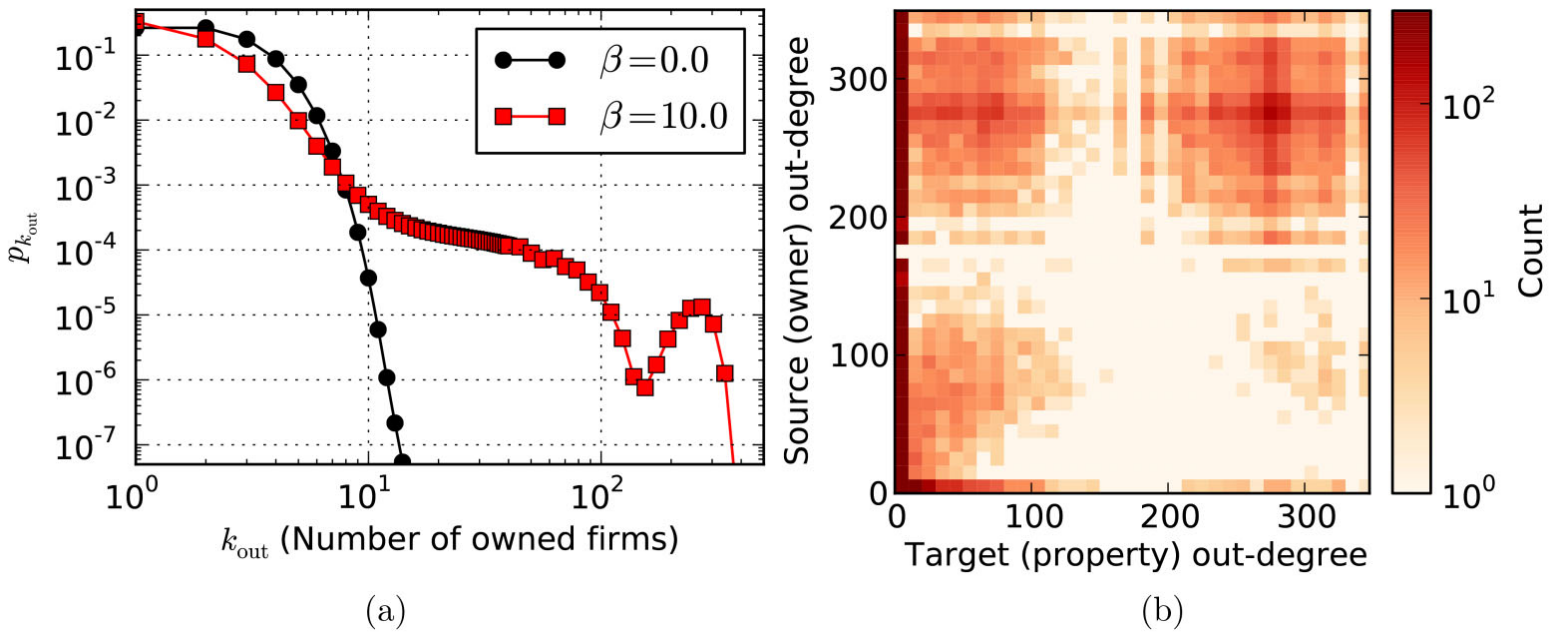
Emergence of a core-perephery structure. (a) Degree distribution of the resulting network for 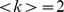, a control propagation value of 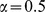, 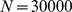 and different values of prior knowledge 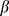; (b) Degree correlation matrix for 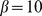, showing the resulting core-periphery structure. See also Fig. 4.

**Figure 4:**
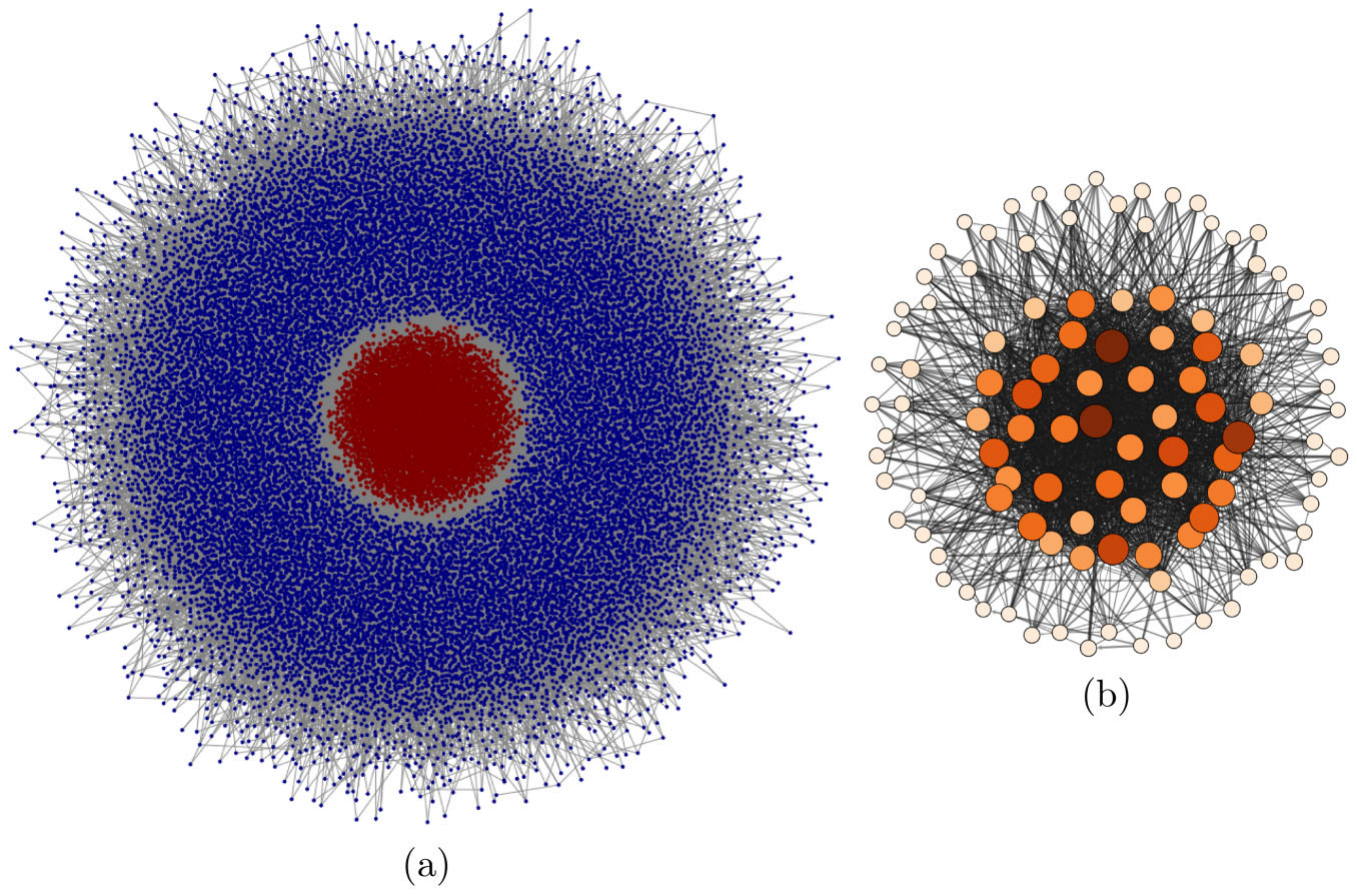
Emergence of a core-perephery structure. (a) Graph layout of the whole network, with red nodes representing a chosen fraction of the most highly connected core, and blue ones the periphery, for 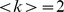, a control propagation value of 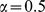, 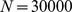 and 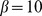; (b) Subgraph of the most powerful companies with 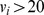 (about 100). The node colors and sizes correspond to the 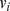 values.

A typical outcome of the dynamics can be seen in Figs. 3 and 4 for a network with 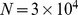 nodes, average degree 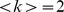, 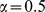, and 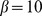 (results for 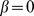 are shown additionally for comparison in (a)), after an equilibration time of about 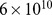 time steps. In contrast to the case with 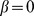, which results in a fully random graph, for a sufficiently high value of 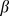 the distribution of firm ownerships (i.e. the out-degree of the nodes) becomes very skewed, with a bimodal form. We can divide the most powerful companies into a broad range which owns shares from 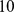 to about 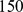 other companies, and a separate group with 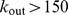. The correlation matrix of this network shows that these high-degree nodes are connected strongly among themselves, and own a large portion of the remaining companies (see Figs. 3 and 4). This corresponds to a highly connected “core” of about 45 nodes with 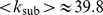, which is highlighted in red in Fig. 4a and can be seen separately in Fig. 4b. The distribution of in-degree (not shown) is bimodal as well with highest values for the inner core. With values up to 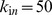, the highest in-degree (number of owners) is considerably below the highest out-degree (number of firms owned at once).

**Figure 5:**
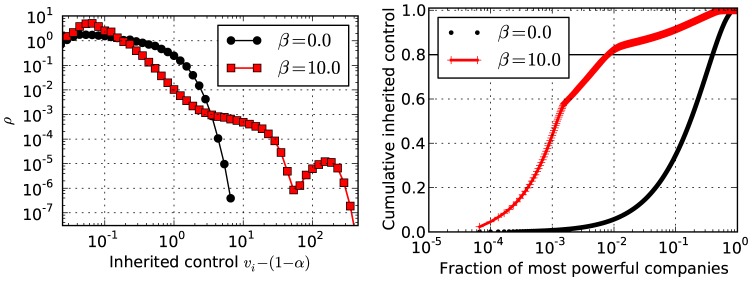
Centralization of control. Results for the same networks as shown in Fig. 2 (a) with 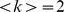, 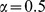, 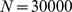 and different values of 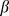. Left: Probability density of inherited control values 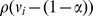; Right: Relative fraction of control as a function of fraction of most powerful companies.

Similarly to the out-degree, the distribution of control values 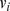 is also bimodal for the network with 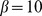 discussed above (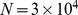, 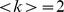 and 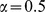), as can be seen in Fig. 5, and is strongly correlated with the out-degree values. The total fraction of companies controlled by the most powerful ones is very large, as shown on the right panel of Fig. 5. For instance, we see that a fraction of around 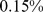 of the central core controls about 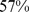 of all companies. The companies with intermediary values of control (and out-degree) also possess a significant part of the global control, e.g. around 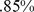 of the most powerful control an additional 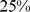 of the network. It is important to emphasize the difference between these two classes of companies for two reasons: Firstly the inner core inherits control from intermediate companies without the need to gather up all the minor companies. In fact the ownership links going out from the inner core (about 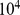) is enough to cover the direct control of only a third of all companies, while the effective control is more than a half. Secondly, the fraction of intermediary companies increases for larger networks. For a network with 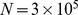 and the same parameter values as above of 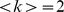, 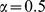, and 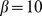, the inner core includes a fraction of only 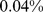, controlling an effective 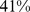 of the total companies. Nonetheless, all the most powerful companies together account for around 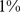 of the network and 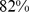 of the total control; values which do not change considerably with system size.

Let us compare the results presented so far with empirical data presented in [9]. For different reasons, this comparison can only be qualitative. First of all, the empirical data includes economic agents with different functions (shareholders, transnational companies and participated companies) out of different sectors (eg. financial and real economy), while we consider identical agents. Secondly, we force every company to be owned 100%, while the empirical data neglects restrained shares and diversified holdings. Thirdly, the control analysis in [9] is done somewhat differently: All the 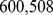 economic agents were considered for the topological characterization, while many companies (80% of all agents there) were neglected for the control analysis. In the empirical data, a strongly connected component of 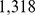 companies controls more than a half of all companies arranged in the out component. This concentration is compatible with the core-periphery structure presented in Fig. 3, however the empirical data does not show a distinct bimodal structure. Nonetheless, there are highly connected substructures in the core, e.g. a structure with 22 highly connected financial companies (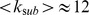) was highlighted in [3]. The control concentration in the empirical data was reported as a fraction of 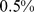 which controls 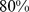 of the network. This is similar to the results of our model (see Fig. 5 on the right). There are, however, features that our model does not reproduce, the most important of which being the out-degree distribution of the network, which in [9] is very broad, and displays no discernible scales, where in our case it is either bimodal or Poisson-like. One possible explanation for this discrepancy is that we have focused on equilibrium steady-state configurations of the dynamics, whereas the real economy is surely far away from such an equilibrium. A more precise model would need to incorporate such transient dynamics in a more realistic way. Nevertheless, the general tendency of the control to be concentrated on relatively few companies is evident in such equilibrium states, and features very prominently in the empirical data as well.

### 2.1 Transition to centralization

**Figure 6:**
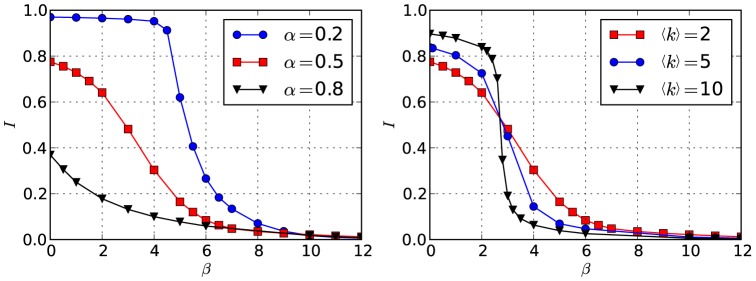
Transition to control centralization. Inverse participation ratio 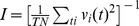 as a function of 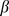, for a network with 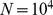, and for (left) 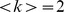 and different values of 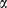 and (right) 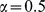 and different values of 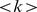.

To investigate the transition from homogeneous non-centralized networks with increasing 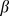, we measured the inverse participation ratio 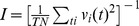 with the time 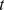 summing over a sufficiently long time window of length 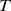 after equilibration. Since 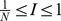, we expect 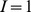 in the perfectly homogeneous case where 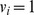 for all nodes, and 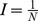 if only one node has 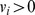, and the control is maximally concentrated. As can be seen in Fig. 6, we observe a smooth transition from very homogeneous companies connected in fully random manner for 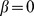, to a pronounced concentration of control for increased 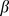, for which the aforementioned core-periphery is observed. Results are shown for networks of size 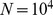 with 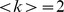 and 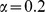, 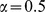 and 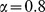 (left), respectively, with 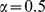 and 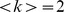, 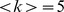 and 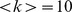 (right). The transition becomes more abrupt when either the average degree 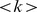 is increased or the parameter 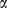 (which determines the fraction of inherited control) is decreased.

**Figure 7:**
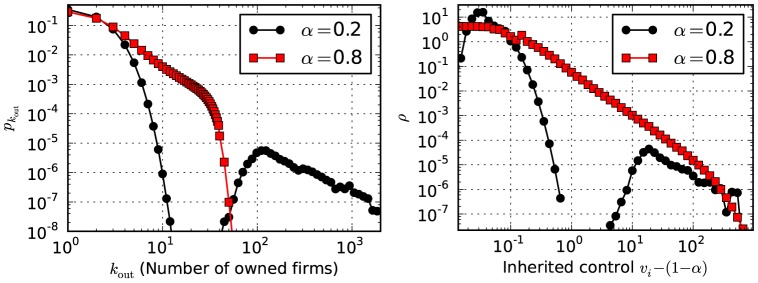
Centralization for different model parameters. Distribution of out-degrees 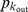 (left) and inherited control 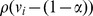 (right) for 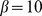, 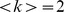 and 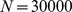 as in Fig. 3 and 5, but for different values of 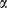.

Centralization of control can emerge in different ways depending on the parameters 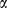 and 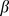. In Fig. 7, it is shown that different values of 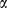 for a high value of 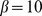 (with 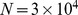 and 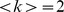 as in Fig. 3 and 5) can lead to a detached controlling core (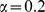) or to broadly distributed control values (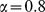). With smaller values of 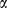, indirect control is suppressed and companies can gain power only by owning large numbers of marginal companies. E.g.: for 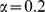, this leads to a highly connected core of 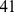 companies having 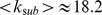, the rest of the companies have very little influence. For larger values of 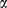, indirect control has a larger effect, which leads to a hierarchical network where companies with small numbers of owned firms 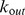 may nevertheless inherit large control values 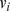. The case with 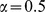 and 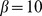 shown in Figs. 3 and 5 exhibits a mixture of these two scenarios. The transition to a centralized core also occurs when increasing 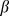 and keeping 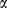 constant (see right panel in Fig. 6).

**Figure 8:**
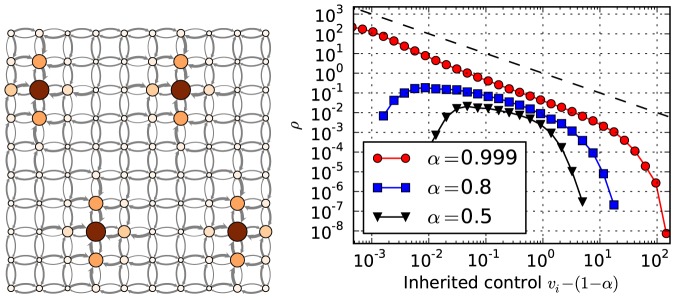
Spontaneous symmetry breaking for static graphs. Left: Graph layout of a 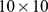 lattice with 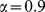. The vertex sizes and colors correspond to the 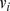 values, and the edge thickness to the 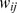 values. Right: Distribution of inherited control 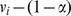 for static Poisson graphs having 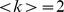 and 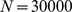, with different values of 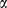 (for 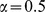 and 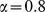 shifted). The dashed line is a power law with exponent 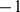.

One interesting aspect of the centralization of control as we have formulated is that it is not entirely dependent on the adaptive dynamics, and occurs also to some extent on graphs which are static. Simply solving Eqs. 1 and 2 will lead to a non-trivial distribution of control values 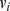 which depend on the (in this case fixed) network topology and the control inheritance parameter 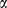. In Fig. 8 is shown on the left the control values obtained for a square 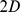 lattice with 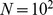 having periodic boundary conditions and bidirectional edges, propagated with 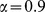. What is observed is a spontaneous symmetry breaking, where despite the topological equivalence shared between all nodes, a hierarchy of control is formed, which is not unique and will vary between each realization of the dynamics. A similar behavior is also observed for fully random graphs, as shown on the right of Fig. 8. Results are presented for static Poisson graphs with 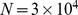, 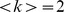 and values of 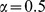, 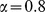, and 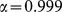. The distribution of control values becomes increasingly broader for larger values of 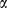, asymptotically approaching a power-law 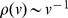 for 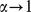. This behavior is similar to a phase transition at 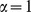, where at this point Eq. 1 no longer converges to a solution.

## Conclusion

We have tested the hypothesis that a rich-get-richer process using a simple, adaptive dynamics is capable of explaining the phenomenon of concentration of control observed in the empirical network of company ownership [9]. The process we proposed incorporates the indirect control that companies have on other companies they own, which increases their buying power in a feedback fashion, and allows them to gain even more control. In our model, the system spontaneously organizes into a steady-state comprised of a well-defined core-periphery structure, which reproduces many qualitative observations in the real data presented in [9], such as the relative portion of control exerted by the dominating companies. Our model shows that this kind of centralized structure can emerge without it being an explicit goal of the companies involved. Instead, it can emerge simply as a result of individual decisions based on local knowledge only, with the effect that powerful companies can increase their relative advantage even further.

It is interesting to note that the topology obtained with our model differs significantly from those resulting from preferential attachment implemented in network growth models, which often lead to scale-free degree distributions [22]–[24]. This type of broad distribution is also present in the empirical network of corporate control [9], [25]. In these growth models condensation is only observed if the preferential attachment is super-linear, which leads to a “winner takes all” situation with central hub composed of a single node [26]. However, our results are compatible with non-growth models with linear preferential attachment, where condensation occurs via a bimodal degree distribution if the edge rewiring rate is sufficiently large [27], [28]. It is also fruitful to compare our model to other agent based models featuring agents competing for centrality. The emergence of hierarchical, centralized states with interesting patterns of global order was reported for agents creating links according to game theory [29]–[31] as well as for very simple effective rules of rewiring according to measured centrality [32], [33]. The stylized model of a society studied in [33] shows a hierarchical structure, if the individuals have a preference for social status. The intuitive emergence of hierarchy is associated with shrinking mobility of single agents within the hierarchy. This effect is present in our model as well and deserves further investigation. Another open question is the effect of a superlinear rich get richer dynamics for 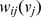 as well as the effect of nonlinearly increasing control with ownership shares (especially high shares above 50% are believed to be connected with highly increasing control). The latter is known to play only a minor role for the real network of corporate control [9] and therefore should not affect the general behavior of the model.

Our results may shed light on certain antitrust regulation strategies. As we found that a simple mechanism without collusion suffices for control centralization, any regulation which is targeted to diminish such activities may prove fruitless. Instead, targeting the self-organizing features which lead to such concentration, such as e.g. limitations on the indirect control of shareholders representing other companies, may appear more promising.
